# Non-invasive detection of divergent metabolic signals in insulin deficiency vs. insulin resistance *in vivo*

**DOI:** 10.1038/s41598-018-20264-w

**Published:** 2018-02-01

**Authors:** Cornelius von Morze, Prasanna K. R. Allu, Gene Y. Chang, Irene Marco-Rius, Eugene Milshteyn, Zhen J. Wang, Michael A. Ohliger, Catherine E. Gleason, John Kurhanewicz, Daniel B. Vigneron, David Pearce

**Affiliations:** 1 Department of Radiology and Biomedical Imaging, University of California, San Francisco, United States; 2 Division of Nephrology, Department of Medicine, University of California, San Francisco, United States

## Abstract

The type 2 diabetic phenotype results from mixed effects of insulin deficiency and insulin resistance, but the relative contributions of these two distinct factors remain poorly characterized, as do the respective roles of the gluconeogenic organs. The purpose of this study was to investigate localized *in vivo* metabolic changes in liver and kidneys of contrasting models of diabetes mellitus (DM): streptozotocin (STZ)-treated wild-type Zucker rats (T1DM) and Zucker diabetic fatty (ZDF) rats (T2DM). Intermediary metabolism was probed using hyperpolarized (HP) [1-^13^C]pyruvate MRI of the liver and kidneys. These data were correlated with gene expression data for key mediators, assessed using rtPCR. Increased HP [1-^13^C]lactate was detected in both models, in association with elevated gluconeogenesis as reflected by increased expression of phosphoenolpyruvate carboxykinase. In contrast, HP [1-^13^C]alanine diverged between the two models, increasing in ZDF rats, while decreasing in the STZ-treated rats. The differences in liver alanine paralleled differences in key lipogenic mediators. Thus, HP [1-^13^C]alanine is a marker that can identify phenotypic differences in kidneys and liver of rats with T1DM vs. T2DM, non-invasively *in vivo*. This approach could provide a powerful diagnostic tool for characterizing tissue metabolic defects and responses to treatment in diabetic patients with ambiguous systemic manifestations.

## Introduction

The characteristic fasting hyperglycemia of diabetes mellitus is due largely to an inability to suppress gluconeogenesis (GNG). In the case of type 1 diabetes (T1DM) the inability is due to insulin deficiency, while in type 2 diabetes (T2DM) it arises from mixed effects of insulin deficiency and/or insulin resistance in gluconeogenic tissues^1,2^. The respective metabolic consequences of these two distinct factors have not been well characterized *in vivo*, nor have the mechanisms underlying these changes. Better understanding of the relative contributions of insulin deficiency and insulin resistance may have important implications for clinical management of T2DM, as intensive glucose control by insulin sensitization and/or supplementation strategies offers at best only modest improvements in disease-related morbidity and mortality^3,4^. Another significant area of uncertainty is the relative contribution of the two major gluconeogenic tissues, liver and kidneys, to total GNG. Although the liver is generally regarded as the dominant source of new glucose production under normal conditions, renal GNG is a major source of glucose in starvation^5^, acidosis^5^, and hepatectomy^6^. There is also evidence that renal GNG contributes importantly to the diabetic phenotype in humans with either T1DM or T2DM^7,8^, as well as in diabetic animals^9–11^. However, assessing organ-specific changes in GNG in humans or animals has required highly invasive approaches, creating a substantial barrier to investigation. Furthermore, measurement of organ-specific GNG *in vivo* is an inherently difficult tracer problem, particularly in kidney, due to the high renal blood flow and correspondingly small changes in tracer concentration, as well as the high capacity of the renal medulla to consume new glucose prior to export.

Systemic manifestations of T1DM and T2DM differ markedly, substantially due to the selective nature of insulin resistance, which impacts the glucoregulatory effects of insulin without interfering with, and possibly even enhancing, other key actions. For example, insulin stimulation of hepatic lipogenesis is often sustained in T2DM, resulting in combined hyperglycemia and hypertriglyceridemia^12^. Recent mechanistic accounts have attributed this effect to selective disruption of the “gluco-suppressive” branch of the insulin signaling pathway^13^, with sustained insulin-dependent stimulation of expression of the lipogenic transcription factor sterol regulatory element binding protein 1c (SREBP-1c) coupled with its activation due to endoplasmic reticulum (ER) stress^14^. Similarly, in the kidney tubules of T2DM patients or animals, even as glucoregulatory insulin signaling declines, insulin stimulation of sodium-retaining pathways is sustained, contributing to salt-sensitive hypertension, a defining feature of the metabolic syndrome, and commonly found in patients with T2DM^15,16^.

Heretofore, there has been no marker that could distinguish T1DM and T2DM at the tissue level non-invasively in live animals. In this study we investigated localized metabolic changes in gluconeogenic tissues of two different models of DM: 1) The Zucker diabetic fatty (ZDF) rat^17^, which develops selective insulin resistance, hyperinsulinemia, and ultimately a highly gluconeogenic, lipogenic, and hypertensive phenotype^18,19^. The adult male ZDF rat has markedly elevated rates of gluconeogenesis as compared with lean controls^20^. Emergence of diabetes in ZDF rats coincides with a striking rise in plasma triglycerides attributable mainly to greatly increased rates of *de novo* lipogenesis^21,22^ in association with increased hepatic levels of the lipogenic transcription factor sterol regulatory element binding protein 1c (SREBP-1c) as well as salt-sensitive hypertension^23^; 2) The insulin-deficient streptozotocin (STZ)-treated model of type 1 diabetes, which also exhibits very high rates of GNG^24^ but in striking contrast to the ZDF rat has markedly suppressed hepatic expression of SREBP-1c and correspondingly low levels of lipogenesis^25^, and does not develop hypertension except as a late manifestation related to kidney damage^26,27^. The metabolic features of these animals, along with WT rats of the same background, were studied *in vivo* using hyperpolarized (HP) ^13^C magnetic resonance imaging (MRI), a powerful new imaging modality for non-invasive metabolic investigations, based on ~50,000-fold nuclear magnetic resonance (NMR) signal enhancements of ^13^C-labeled substrates via dissolution dynamic nuclear polarization (DNP)^28^. This technique features the unique capability to track localized enzymatic conversions through key biochemical pathways in real time based on resonance frequency differences among individual metabolites^29^.

Based on its excellent polarization characteristics and role as a key metabolic intermediate, HP [1-^13^C]pyruvate has been widely applied to investigate localized changes in intermediary glucose metabolism in multiple diseases, and has recently been successfully translated into studies of human subjects^30,31^. However, there have been relatively few studies investigating diabetes *per se*^32–36^. Notably, two recent studies described large changes in the enzymatic conversions of HP ^13^C signal through reactions catalyzed via lactate dehydrogenase (LDH), alanine transaminase (ALT), and pyruvate dehydrogenase (PDH) in rat liver and kidneys in fasting and insulin deficient diabetic states^32,36^. In one study, these changes were shown to parallel changes in gene expression of phosphoenolpyruvate carboxykinase (PEPCK) in both liver and kidney of STZ-treated rats^36^. However, it remains unknown if metabolic conversions detectable by HP MRI differ or are the same in models of T1DM vs. T2DM. Furthermore, there is relatively little known regarding the relative contributions of kidney and liver to these two different types of DM. We therefore directly compared [1-^13^C]pyruvate conversions *in vivo*, in the above-described models of T1 and T2DM. Results were interpreted in comparison with results of transcriptional analysis of both gluconeogenic and lipogenic pathways from snap-frozen liver and kidney tissue samples taken from the same animals.

## Results

### HP metabolite profiles of STZ-treated wild type and obese ZDF rats

**Figure 1:**
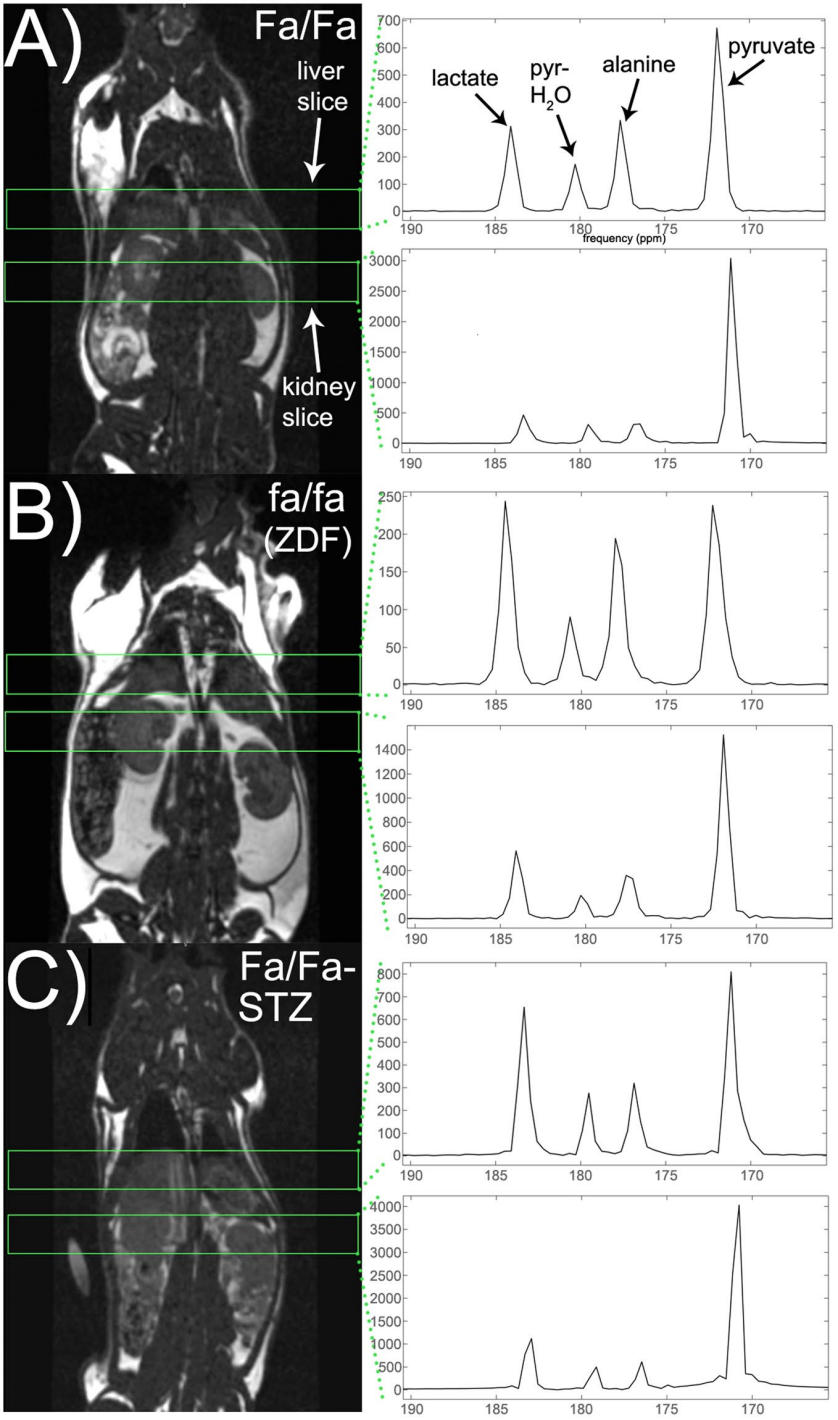
Localized MR spectra of HP [1-^13^C]pyruvate from individual Zucker rats sampled from the subgroups described in text: wild type Zucker (*Fa/Fa*) (**A**), obese Zucker (*fa/fa*) (**B**), and wild type Zucker treated with STZ and subsequent insulin withdrawal for 24–36 h (*Fa/Fa-STZ*) (C). Resonances corresponding to [1-^13^C]lactate (184 ppm), [1-^13^C]pyruvate hydrate (180 ppm), [1-^13^C]alanine (177 ppm), and [1-^13^C]pyruvate (172 ppm) are easily identified in all spectra (arrows). HP ^13^C spectra are shown to the right of standard coronal anatomic ^1^H MRI images indicating positions of slices for ^13^C MR data acquisition (green rectangles). In each rat, spectra were acquired from each of two adjacent 12-mm thick axial slices through the liver and kidneys, respectively. MR spectra were summed over all acquisition time points over the 30-second acquisition window, and scaled to the approximate spectral noise level (i.e. the MR data has units of signal-to-noise ratio).

**Figure 2:**
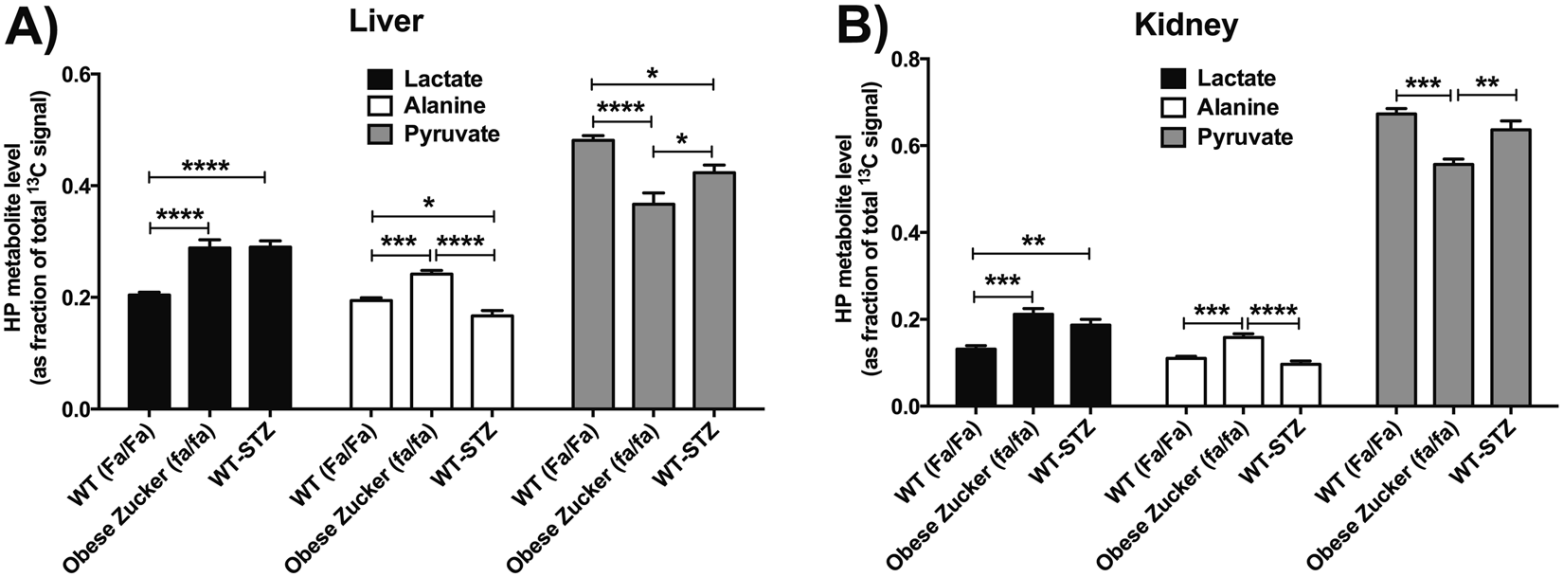
Quantitative summary of the results of localized MR spectroscopy of HP [1-^13^C]pyruvate in liver (**A**) and kidney (**B**) of Zucker rats, grouped by metabolite. Each metabolite / organ group includes results from three experimental groups as described in text: wild type (WT, *Fa/Fa*, n = 7), diabetic obese Zucker (*fa/fa*, n = 6), and diabetic WT-STZ (*Fa/Fa-STZ*, n = 6) rats. HP metabolite levels are expressed as a fraction of the total ^13^C signal in the corresponding spectrum. Group means are shown with error bars indicating S.E.M. Statistically significant comparisons from one-way ANOVA are labeled (*p < 0.05, **p < 0.01, ***p < 0.001, ****p < 0.0001).

Intravenously injected HP [1-^13^C]pyruvate was rapidly metabolized to [1-^13^C]lactate and [1-^13^C]alanine in the liver and kidneys of Zucker rats from all groups (all male, age ~12 weeks). Basic rat physiologic parameters at time of imaging are shown in Supplementary Figure S1. Notably, ZDF rats were significantly heavier than the other groups and had much greater adipose tissue volume (as estimated by ^1^H MRI). Blood glucoses of both STZ-treated WT rats and ZDF, sampled at time of imaging, were also elevated relative to controls. HP ^13^C MR spectra from each experimental group are shown in Fig. 1. As depicted in the quantitative summary of results (Fig. 2), conversion of injected HP [1-^13^C]pyruvate to [1-^13^C]lactate, represented as the fraction of HP [1-^13^C]lactate signal / total ^13^C signal, was higher in both liver and kidneys of STZ-treated rats (*Fa/Fa-STZ*: liver + 42%, *p* < 0.0001; kidney + 42%, *p* < 0.01), and ZDF rats (*fa/fa:* liver + 41%, *p* < 0.0001; kidney + 61%, *p* < 0.001), as compared to wild type age- and sex-matched wild type Zucker rats (*Fa/Fa*). Statistical results were derived from one-way ANOVA between groups by organ, with Tukey correction for multiple comparisons (ANOVA summary statistics for lactate in liver: *p* < 0.0001, *F* = 22.65, *df* = 16; kidney: *p* < 0.001, *F* = 13.53, *df* = 16). Although not specific for cytoplasmic NAD(H), colorimetric assays for whole cell NAD(H) content (Supplementary Figure S2) likewise revealed trends toward increased NADH levels in kidneys and liver of both *Fa/Fa-STZ* and *fa/fa* diabetic rats, consistent with the increased reducing power required to support gluconeogenesis^37–39^ as well as conversion of HP pyruvate to lactate. The especially large increases in NADH content in *fa/fa* rats are attributable to increased TCA cycle flux in T2DM. Also consistent with this interpretation was a divergence noted in the measured NAD^+^/NADH ratios between *Fa/Fa-STZ* and *fa/fa* rats.

### HP alanine signals diverge in type 1 and type 2 diabetes models

In contrast to the metabolic conversion of HP [1-^13^C]pyruvate to [1-^13^C]lactate, conversion of HP [1-^13^C]pyruvate to [1-^13^C]alanine (represented as the fraction of HP [1-^13^C]alanine / total ^13^C signal), diverged between the two models of diabetes in both kidney and liver. Consistent with earlier results obtained in STZ-treated Sprague-Dawley rats^36^, this fraction was lower in STZ-treated rats than in wild type controls (*Fa/Fa*) (*Fa/Fa-STZ*: liver −14%, *p* < 0.05; kidney -12%, n.s.). However, in marked contrast, the HP [1-^13^C]alanine / total ^13^C signal was significantly higher in insulin resistant ZDF rats than in controls (*fa/fa:* liver +24%, *p* < 0.01; kidney +44%, *p* < 0.001). The difference between the obese ZDF group and the STZ-treated WT group was highly significant (*fa/fa*: liver +45%, p < 0.0001; kidney + 64%, p < 0.0001). Here again statistical results were derived by group-wise one-way ANOVA comparison with Tukey correction (ANOVA summary statistics for alanine in liver: *p* < 0.0001, *F* = 26.69, *df* = 16; kidney: *p* < 0.0001, *F* = 21.20, *df* = 16).

**Figure 3:**
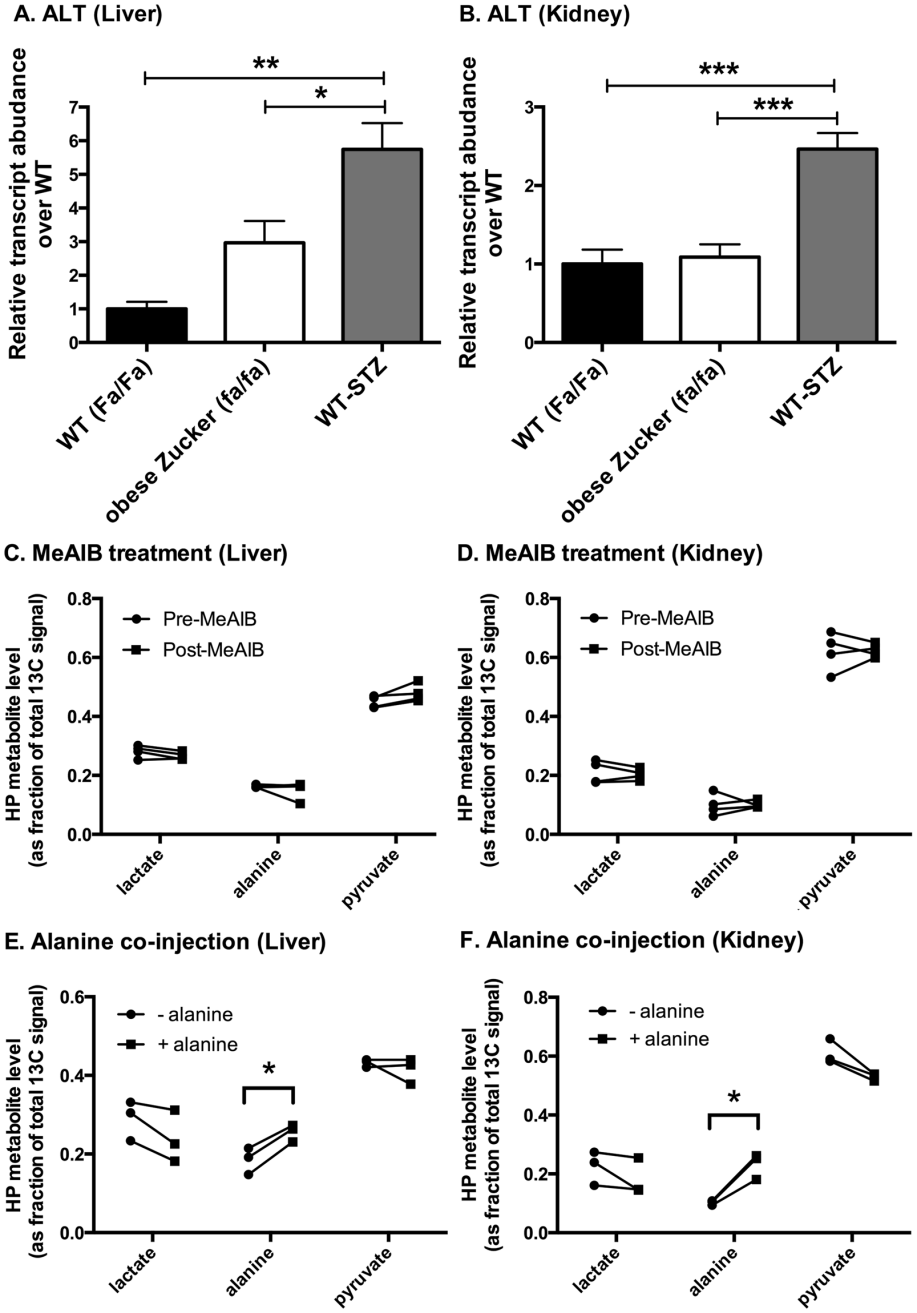
Investigation of factors that could influence the HP alanine signal level. *Panels A&B:* rtPCR measurements of ALT expression in liver (**A**) and kidney (**B**) tissues of age and sex matched WT (Fa/Fa) (n = 4), obese Zucker (fa/fa), (n = 9) and WT-STZ (n = 6) rats. *Panels C&D:* Effect of amino acid transport system A inhibition (1000 mg/kg i.v. MeAIB treatment, 90 minutes prior to “post-MeAIB” data point) on metabolites of HP [1-^13^C]pyruvate in liver (**C**) and kidney (**D**) of normal fed Sprague Dawley rats (n = 4). *Panels E&F:* Effect of co-injection with HP [1-^13^C]pyruvate of equimolar unlabeled alanine, on HP metabolite signals detected in liver (**E**) and kidney (**F**) of normal fed Sprague Dawley rats (n = 3). For PCR, the fold changes were measured relative to WT (Fa/Fa) and calculated with 2^−ΔCt^ method. The data were normalized to β-actin. All PCR data represent mean+/−S.E.M. from three independent experiments, and statistical analysis was performed by one-way ANOVA with Tukey correction for multiple comparisons. (*p < 0.05, **p < 0.01, ***p < 0.001) For C–E, individual metabolites were compared using paired, two-tailed t-tests. (*p < 0.05).

While an increasing rate of gluconeogenesis is coupled to reduction of the cytoplasmic NAD(H) system^37–39^, likely explaining the parallel increases in HP lactate signals in both diabetes models, we were intrigued by the apparently divergent HP alanine signal levels in the type 1 vs. type 2 models. Initial hypotheses that these changes could be related to changes in ALT expression or changes in alanine transport were discounted through further experiments. First, although there were differences in ALT expression level (Fig. 3A and B) among the groups, these differences did not parallel the divergent changes in HP alanine signal. Second, inhibition of alanine transport by the system A amino acid transport inhibitor α-(methylamino)isobutyrate^40,41^ (MeAIB) produced no discernible changes in the HP liver or kidney spectra of normal fed Sprague Dawley rats (Fig. 3C and D), suggesting that variations in alanine transport are unlikely to account for changes in HP alanine signals. Interestingly, co-injection of equimolar quantities of unlabeled alanine along with HP [1-^13^C]pyruvate resulted in a large increase in the HP alanine signal in both liver and kidney of normal fed Sprague Dawley rats (Fig. 3E and F), suggesting that the HP alanine signal is dominated by isotope exchange flux as opposed to true metabolic flux of pyruvate to alanine, and thus depends largely on the cellular alanine pool size accessible to injected HP pyruvate and ALT. In contrast, co-injection of unlabeled α-ketoglutarate produced no effects on the HP spectra (Supplementary Figure S3), also consistent with the hypothesis that the HP alanine signal mainly reflects the cellular alanine level. This result is similar to the HP lactate signal, which is known to depend largely on lactate pool size^42,43^, based on analogous considerations for LDH, which has similarly high bidirectional activity as ALT.

### Gluconeogenic and lipogenic enzyme expression profiles

**Figure 4:**
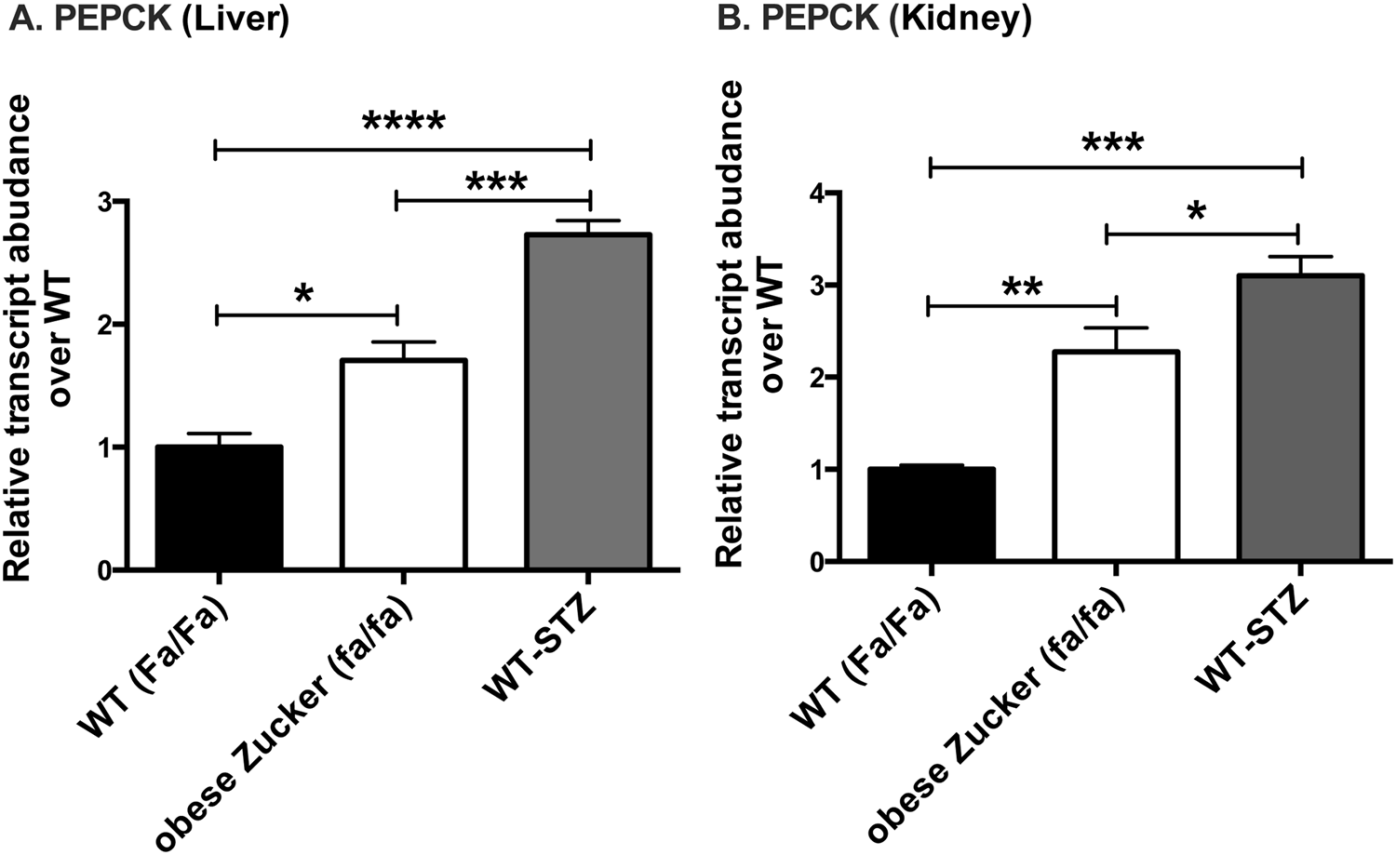
rtPCR PEPCK expression measurements in liver (**A**) and kidney (**B**) of age and sex matched WT (Fa/Fa) (n = 4), obese Zucker (fa/fa) (n = 9), and WT-STZ (n = 6) rats. The fold changes were measured relative to WT (Fa/Fa) and calculated with 2^−ΔCt^ method. The data were normalized to β-actin. All data represent mean+/−S.E.M. from three independent experiments. Statistical analysis was performed by one-way ANOVA with Tukey correction for multiple comparisons. (*p < 0.05, **p < 0.01, ***p < 0.001, ****p < 0.0001).

To help interpret the above substrate-level changes in the context of the regulation of gluconeogenesis and lipogenesis, we next measured the expression of several key gluconeogenic and lipogenic enzymes and transcription factors in the liver and kidney of all three experimental groups profiled by HP [1-^13^C]pyruvate MRI. PEPCK catalyzes a key rate-limiting step in GNG, and is mainly regulated at a transcriptional level. We therefore used PEPCK expression as a tissue marker of changes in GNG. As expected, PEPCK expression was upregulated in both diabetes models in both liver and kidneys (Fig. 4). In liver, obese Zucker (*fa/fa*) and WT-STZ showed 1.7-fold (*p* < 0.05) and 2.7-fold (*p* < 0.0001) increases as compared to WT (*Fa/Fa*) (one-way ANOVA summary statistics: *F* = 27.42, *p* < 0.0001, *df = *14). In kidney, obese Zucker (*fa/fa)* and WT-STZ showed 2.27-fold (*p* < 0.01) and 3.1-fold (*p* < 0.001) increases as compared with WT (*Fa/Fa*) (one-way ANOVA summary statistics: *F* = 15.55, *p* < 0.001, *df* = 15). Western blots for PEPCK using extracts from kidney and liver tissues of rats from each experimental group yielded similar results (Supplementary Figure S4), providing strong evidence that changes in PEPCK mRNA level do result in changes in protein content, consistent with prior literature^44^.

**Figure 5:**
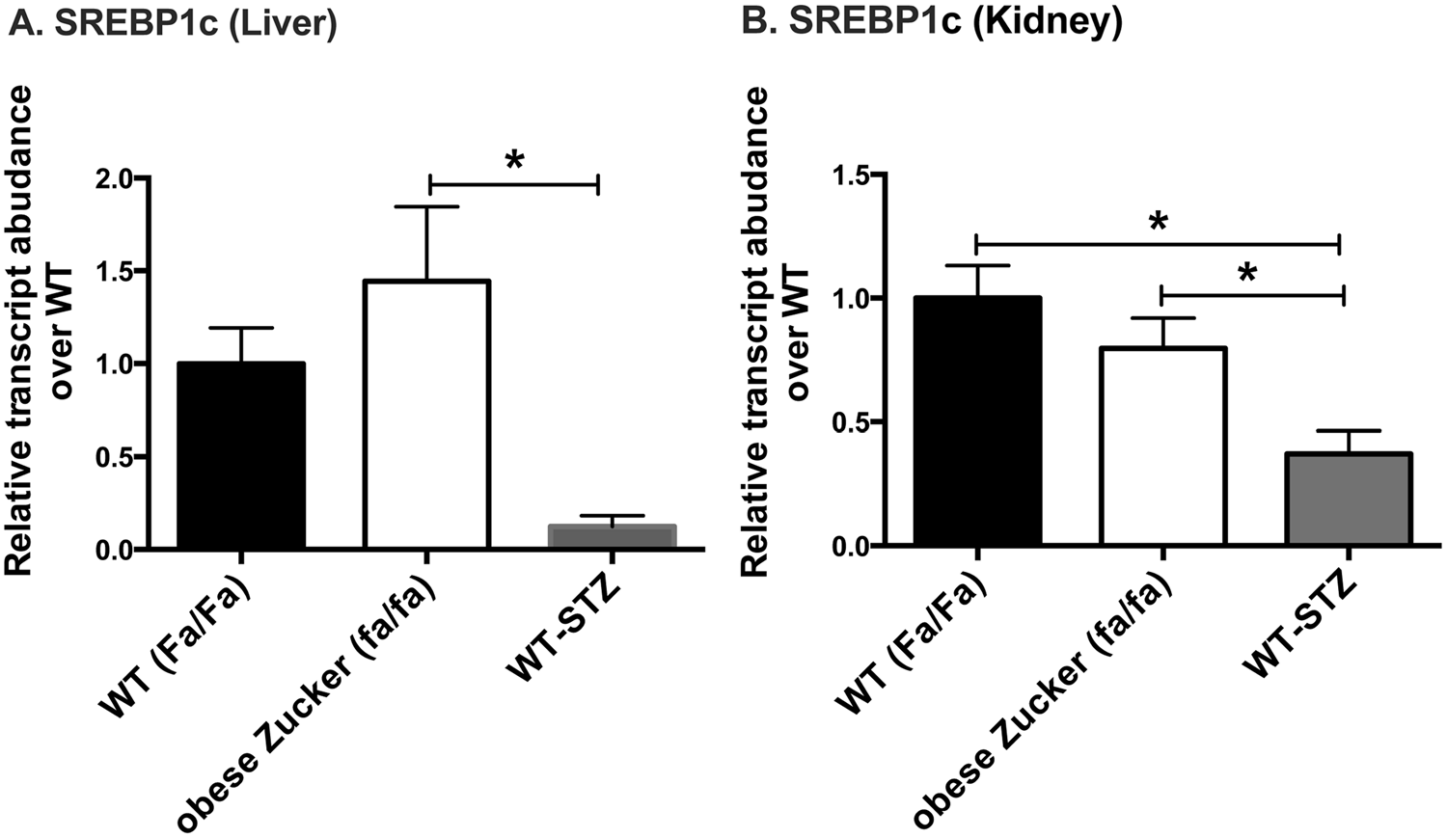
Hepatic (**A**) and renal (**B**) expression levels of SREBP-1c measured using rtPCR. Measurements were obtained in liver and kidney tissue samples from age and sex matched WT (Fa/Fa) (n = 4), obese Zucker (fa/fa) (n = 9), and WT-STZ (n = 6) rats. The fold changes were measured relative to WT (Fa/Fa) controls and calculated by 2^−ΔCt^ method. The data were normalized to β-actin. All data represent mean+/−S.E.M. from three independent experiments. Statistical analysis was performed by one-way ANOVA with Tukey correction for multiple comparisons. (*p < 0.05).

**Figure 6:**
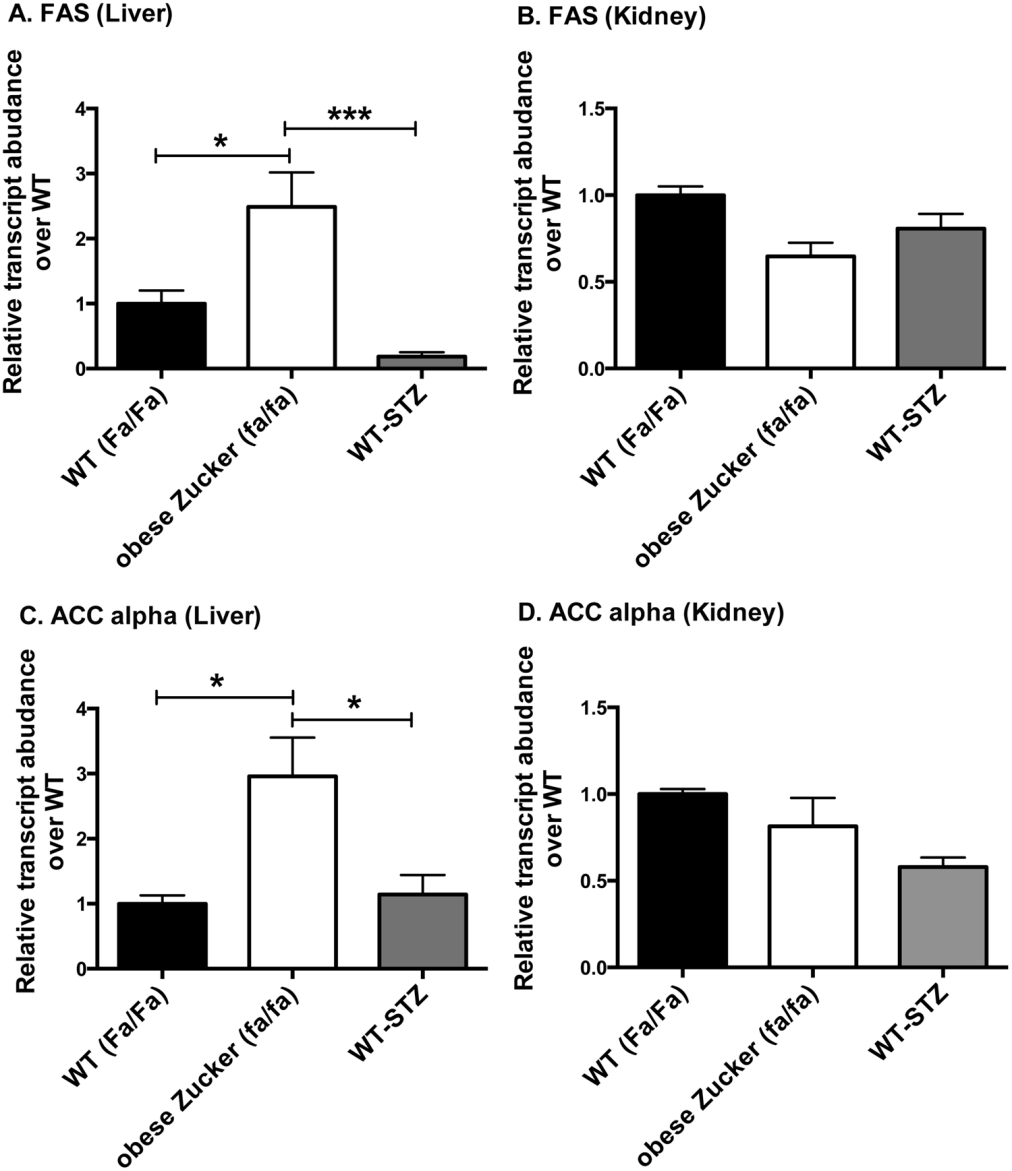
Hepatic (**A**,**C**) and renal (**B**,**D**) expression levels of lipogenic enzymes measured using rtPCR: fatty acid synthase (FAS) (**A**,**B**), acetyl-CoA carboxylase alpha (ACC alpha) (**C**,**D**). Measurements were obtained in liver and kidney tissue samples from age and sex matched WT (Fa/Fa) (n = 4), obese Zucker (fa/fa) (n = 9) and WT-STZ (n = 6) rats. The fold changes were measured relative to WT (Fa/Fa) controls and calculated by 2^−ΔCt^ method. The data were normalized to β-actin. All data represent mean + /−S.E.M. from three independent experiments. Statistical analysis was performed by one-way ANOVA with Tukey correction for multiple comparisons. (*p < 0.05, ***p < 0.001).

Expression levels of lipogenic master regulator SREBP-1c (Fig. 5) on the other hand differed markedly between the two diabetes models in both liver and kidneys. SREBP-1c expression was 11.7-fold higher in the liver (*p* < 0.05) and 2.7-fold higher in the kidney (*p* < 0.05) of obese Zucker (*fa/fa*) rats as compared to STZ-treated WT controls (one-way ANOVA summary statistics for SREBP-1c in liver: *F* = 4.709, *p* < 0.05, *df* = 15; kidney: *F* = 6.23, *p* < 0.05, *df* = 15). In the liver only, the differences in SREBP-1c were accompanied by differences in target lipogenic enzymes, fatty acid synthase (FAS) and acetyl-CoA carboxylase alpha (ACC alpha) (Fig. 6). Hepatic expression levels of FAS and ACC alpha, respectively, were 13.4-fold (*p* < 0.001) and 2.6-fold (*p* < 0.05) greater in obese Zucker (*fa/fa*) rats than in STZ-treated WT rats (ANOVA summary statistics for liver FAS: *F* = 11.63, *p* < 0.01, *df* = 13; ACC: *F* = 5.67, *p* < 0.05, *df* = 14). Interestingly, expression of liver-type pyruvate kinase (LPK) (Supplementary Data Figure S5), which opposes the action of PEPCK and is also integral to lipogenesis from glucose, also trended up in liver but not kidney of the obese Zucker rats (2.8-fold increase over WT-STZ, n.s.) (one-way ANOVA summary statistics for LPK in liver: *F* = 3.66, *p* = 0.058, *df* = 12). No significant change was detected in either hepatic or renal expression of ChREBP (Supplementary Data Figure S6).

## Discussion

Distinct characteristic modulations of the enzymatic conversions catalyzed by LDH and ALT were detected in HP [1-^13^C]pyruvate MR spectra of gluconeogenic tissues of Zucker rat models of type 1 (insulin-deprived STZ-treated wild type) and type 2 (ZDF) diabetes. Consistent with earlier results in Sprague-Dawley rats^36^, the increased HP lactate signal in the liver and kidneys of both STZ-treated WT Zucker and ZDF rats appears to reflect increased rates of gluconeogenesis in these organs. To facilitate the synthesis of glucose, GNG requires the generation of cytosolic reducing power in the NAD(H) system^45^, which is normally very limited (normal free NADH/NAD^+^_cytoplasm_~1/700). While other mechanisms could also increase the lactate level, increased rates of gluconeogenesis are coupled to an elevation of the cytoplasmic NADH/NAD^+^ ratio^37–39^ and thus higher lactate levels, likely explaining the HP lactate results in this study. Colorimetric assays of whole cell NAD(H) content were consistent with these hypotheses. It must however be noted that these measurements were likely dominated by a mitochondrial component which is not directly relevant to the LDH reaction, which is generally considered to be compartmentalized to cytoplasm only. Furthermore, such measurements cannot distinguish between free and bound forms of co-factor, of which only the free form sets the equilibrium between pyruvate and lactate levels. The divergence in whole cell NADH/NAD^+^ ratio between WT-STZ and fa/fa rats may reflect changes in TCA cycle flux between these conditions.

While the HP lactate level was found to be elevated in both types of diabetes, the HP alanine level clearly diverged between the two models, decreasing in the type 1 model and increasing in the type 2 model. HP alanine signal appears to largely reflect the endogenous level of cellular alanine, as co-injection of unlabeled alanine with HP [1-^13^C]pyruvate similarly increased the HP alanine signal level. ALT expression does not appear to control the HP alanine level, as the only detected difference in ALT expression level was an elevation in STZ-treated rats with respect to all other groups, consistent with prior reports^46^. Since co-injection of unlabeled alanine greatly raised the HP alanine level in normal rats, even the basal expression of ALT (i.e. no STZ injection) appears sufficient to establish sufficient exchange so that the alanine level exerts larger control over HP alanine signal than the ALT expression level. We have previously observed a graded decline in liver and kidney HP alanine levels among normal fed, fasting, and STZ-diabetic Sprague Dawley rats^36^, and this effect of STZ on HP alanine levels was reproduced here in wild type Zucker rats. In this study we furthermore detected a large *elevation* of the HP alanine level in ZDF rats (*fa/fa*) as compared with wild type Zucker rats (*Fa/Fa*). Prior studies have also suggested changes in liver alanine content in diabetes, but were limited by destructive methodology. Large reductions in alanine levels (~50%) were measured in freeze-clamped livers of fasting and alloxan-based insulin-deficient rat models of diabetes^47^, which are similar to the STZ model used in this study. Increased liver alanine content was also suggested by measurements in ZDF rats (~36%) with respect to lean controls, although this observation was based on a limited set of measurements^48^. Such changes in tissue alanine content are not necessarily paralleled in blood since tissue alanine is maintained in a concentration gradient with blood, even though alanine is a mobile amino acid. A prior study in humans found no significant changes in blood alanine content between normal and type 2 diabetic patients^7^.

Changes in HP alanine signal between the two models of diabetes paralleled expression of the lipogenic transcription factor SREBP-1c, leading to higher expression of the lipogenic enzymes FAS and ACC alpha in the liver of ZDF rats. Hepatic LPK expression also trended upward in the ZDF liver (but not kidney), consistent with its integral role in lipogenesis from glucose. Balance between LPK and PEPCK is also a key regulatory point in GNG. The “rheostat” functionality of LPK could be impaired in obese Zucker rats (*fa/fa*) due to the significantly higher alanine levels observed, which strongly inhibit LPK allosterically (e.g. 50% inhibited at 0.1 mM alanine)^49^. Potentiating LPK by treatment with the fructose analog 2,5-anhydro-D-mannitol^50^ greatly attenuates the hyperglycemia of obese diabetic *db/db* mice (i.e. with an equivalent leptin receptor defect) but not their STZ-treated wild type controls^51^.

Given the striking phenotypic difference between the two models in terms of hepatic lipogenesis, it is tempting to speculate that for liver, the differences in alanine signal are mechanistically related to the differences in lipogenesis. One possibility is the transmission of an effect through shared biochemical equilibria among major enzyme systems, which modulate the cellular level of many metabolites. The cytosolic NADP(H) system, which supplies reducing power for lipogenesis, is maintained in a much more reduced state than the NAD(H) system (~100,000 × more reduced) by a near-equilibrium network of highly active cytosolic dehydrogenases and transaminases that depress cytosolic NADH and NADP^+^ to low levels^52^. ALT is part of one pathway that allows NADH to reduce NADP^+^ extensively, via isocitrate dehydrogenase-1 (IDH1). Changes in this system could thus potentially be transmitted to alanine. Another relationship is the common involvement of activated vitamin B6 co-factor pyridoxal phosphate in both alanine transamination and sphingolipid metabolism, which is strongly implicated in the development of hepatic insulin resistance. Alterations in vitamin B6 synthesis, metabolism, and/or trafficking could produce parallel effects on transamination and fatty acid metabolism^53^. In the case of the type 1 diabetic rats, the drop in alanine level is likely related to depletion of substrate due to diversion to GNG, analogous to an enhanced version of fasting^36^.

However, it is notable that renal alanine signal was similarly distinct between ZDF and STZ models, yet there were no significant differences in lipogenic enzymes in this organ. This result is consistent with the relatively low level of lipogenesis in the kidneys in general^54^. Importantly, other parameters of renal physiology do diverge between insulin deficient and insulin resistant animals. Salt avidity is higher in insulin resistant than in insulin deficient states, which plays an important role in the early onset of hypertension in T2DM^15^. Regardless of the mechanism, HP alanine signal is a strong marker that distinguishes type 1 and type 2 diabetic character in both kidney and liver of Zucker rats.

The large increases in HP lactate signal measured in the diabetic kidneys, and corresponding increases in expression of PEPCK, are consistent with an important renal contribution to the abnormal GNG of diabetes. In support of this interpretation, we also previously measured a 2.7-fold increase in the expression of PEPCK in the kidneys of Sprague Dawley rats subjected to STZ treatment (as compared to a 5.9-fold increase in liver)^36^. PEPCK expression is frequently interpreted as a surrogate marker for GNG^44^, and controls a rate limiting step in GNG. Absence of induction of renal LPK in ZDF rats, and high renal HP alanine levels that may inhibit LPK allosterically^49^, are also consistent with an important renal contribution to GNG in T2DM. Acidosis can also cause a well-characterized stimulation of renal GNG^5^. However, we have not observed significant ketosis (e.g. blood ketones > 2 mM) in our STZ model with insulin withdrawal period of 24-36 hours, based on measurements of β-hydroxybutyrate in blood using a consumer ketone meter (Nova Biomedical, Waltham, MA). Alternative explanations for the elevated renal HP lactate signal in the diabetic rat include direct injury via STZ, although no such type of metabolic effect has been reported^55^, and furthermore such an effect could not account for this observation in the type 2 model.

In conclusion, we have identified metabolic differences in gluconeogenic tissues between models of T1 and T2DM, which are reflected in the intermediary metabolism of HP [1-^13^C]pyruvate detected non-invasively, as well as in the expression profiles of key mediators. Multiple non-invasive diagnostic tools are already available for diabetes, based largely on analysis of serum and/or urine samples. In our view, the added value of non-invasive HP ^13^C MRI in this context is the much greater insight provided into tissue-specific metabolic changes in diabetes, which have previously remained obscure. We envision that this insight could ultimately be applied clinically to characterizing tissue metabolic abnormalities, particularly in the context of ambiguous forms of DM, or metabolic responses to novel therapeutic agents.

## Methods

### Zucker model

The Zucker diabetic fatty (ZDF) rat is a leptin receptor-deficient model of obesity-related type 2 diabetes, which was developed by selective inbreeding for diabetic traits of Zucker fatty rats carrying the spontaneous *fa* mutation of the leptin receptor gene LEPR, which displays Mendelian recessive inheritance^17^. While lean rats of the ZDF line (*Fa/Fa* or *Fa/fa*) are phenotypically normal, the obese rat (i.e. ZDF or *fa/fa*) consistently recapitulates many features of type 2 diabetes in obese humans, including hyperinsulinemia, hyperlipidemia, and hyperglycemia with associated complications over time^18,56^. Seven wild-type (WT) male Zucker rats (*Fa/Fa*), six obese male Zucker rats (*fa/fa*), and six STZ-treated WT male Zucker rats were scanned. Rats were scanned at 12 ± 1 weeks of age (diabetic stage). Mutational status of individual littermates was determined by genotyping via restriction fragment length polymorphism (RFLP) analysis. All animal studies described in this paper were conducted in accordance with all relevant guidelines and regulations, and in accordance with a protocol approved by the UCSF Institutional Animal Care and Use Committee (UCSF IACUC, protocol #AN108035).

### STZ model

Streptozotocin (STZ) was administered as a single dose intraperitoneal injection (60 mg/kg) (Sigma Aldrich, St. Louis, MO) to ablate pancreatic beta cells, and these rats were confirmed diabetic by blood glucose measurement at 48 h after injection. STZ-treated rats were given daily insulin injections to maintain normal metabolic status until 24-36 h prior to the scanning session.

### *In vivo* experiments

Nineteen HP ^13^C MRI scanning sessions were conducted. All obese Zucker rats and STZ-treated WT Zucker rats were diabetic by the time of scanning (blood glucose > 450 mg/dL). Blood glucose levels were measured at time of scan using a consumer glucometer (Bayer Healthcare, Mishawaka, IN). All rats were kept on normal chow diet with unlimited access to food and water at all times. At the time of imaging, each rat was anesthetized using inhalational isoflurane (1.5% via nose cone, flow rate 1 L/min), with lateral tail vein catheter implanted just prior to placement in the scanner.

### Dissolution dynamic nuclear polarization

[1-^13^C]pyruvate was polarized by dissolution dynamic nuclear polarization (DNP) as described in previous studies^36^. Briefly, pure [1-^13^C]pyruvic acid ( ≥ 99% enriched, Sigma ISOTEC, Miamisburg, OH) was mixed with 15 mM trityl radical OX063 and 1.0 mM Gd-DOTA (Guerbet, Roissy, France), and then polarized in a commercial HyperSense polarizer (Oxford Instruments, Tubney Woods, UK) operating at 3.35 Tesla, 94.1 GHz, and 1.3 K, for a period of approximately one hour. The polarized sample was rapidly dissolved in NaOH/Tris buffer to attain a neutral, room temperature 80 mM HP [1-^13^C]pyruvate solution, which was rapidly transported to the MRI scanner for injection.

### Hyperpolarized MR experiments

Scans were conducted in a 3 Tesla clinical MRI scanner equipped with multi-nuclear ^1^H/^13^C capability (GE Healthcare, Waukesha, WI). Rats were imaged in a custom dual-tuned ^1^H/^13^C quadrature insert transceiver volume coil. For each HP experiment, 2.2 mL 80 mM HP [1-^13^C]pyruvate solution was injected into each rat, over 12 s. The scan consisted of dynamically alternating slab-selective MR spectroscopic acquisition of a pair of adjacent axial slabs through the liver and kidneys, respectively (slab thickness = 12 mm, spacing = 5-10 mm depending on rat anatomy), starting 20 s after the start of injection and repeating every 3 s over the course of 27 s, for a total of 10 scans. The flip angles of the scans were gradually increased over the course of the acquisition using a recursive relationship in order to fully expend the HP magnetization^57^. Anatomic 3D SSFP ^1^H images were also acquired for anatomic reference and semi-automated estimation of adipose tissue volume.

### Amino acid transport inhibition

To exclude potential effects of modulation of amino acid transport on HP signal levels, four normal non-diabetic male Sprague-Dawley rats (~400 g) were scanned before and after inhibition of the A amino acid transport system by α-(methylamino)isobutyrate^40,41^ (MeAIB), in a normal fed state. In this case, 1000 mg/kg MeAIB^58^ (Sigma Aldrich, St. Louis, MO) was injected intravenously 15 minutes after a baseline HP [1-^13^C]pyruvate scan, and a repeat HP [1-^13^C]pyruvate study was performed approximately 90 minutes after the MeAIB injection. Multiple prior studies have established the similarity of MR data obtained from repeat injections of HP [1-^13^C]pyruvate in rats, when separated by intervals on the order of an hour (or even less)^59^.

### Hyperpolarized MR experiments with co-injection of unlabeled products of the ALT reaction

To probe the influence of pool size effects on HP metabolic imaging results, unlabeled products of the ALT reaction system (*L*-alanine or α-ketoglutarate) were co-injected together with HP [1-^13^C]pyruvate, in equimolar quantities in three Sprague Dawley rats. To compensate for the potentially slower cellular uptake of these molecules, which cannot use monocarboxylate (MCT) transporters, these products were injected approximately one minute prior to acquisition of the HP MR data. Each experiment was conducted after a baseline scan using HP [1-^13^C]pyruvate only, approximately 90 minutes prior.

### RNA extraction and quantitative real-time PCR

Liver and kidneys were harvested from four wild-type (WT) male Zucker rats (*Fa/Fa*), nine obese male Zucker rats (*fa/fa*), and six STZ-treated WT male Zucker rats. After acquisition of MRI scans, liver and kidneys were harvested and snap frozen in liquid nitrogen, and animals were euthanized. Frozen liver and kidney tissues were powdered using mortar and pestle, and RNA was extracted using the RNeasy lipid tissue Mini kit (Qiagen, Germantown, MD) for liver samples and RNeasy Mini kit (Qiagen) for kidney samples according to the manufacturer’s instructions. RNA yield was detected using Nano Drop Spectrophotometer (ND-1000, version 3.8.1) (Thermo Scientific, Waltham, MA). Complementary DNA (cDNA) was Reverse transcribed with 1 μg total RNA using iScript cDNA synthesis kit (Bio-Rad, Hercules, CA). Gene-specific primers were designed according to known rat sequences and shown in Table 1. For quantitative measurement of specific messenger RNA (mRNA) 1 μL of cDNA generated by reverse transcription was added to 9 μL of PCR mix containing iTaq Universal SYBER Green Super mix (Bio-Rad) 0.4 μM primers, and RNAse free water. The real-time PCR cycling conditions were denaturation at 95 °C for 15 s, annealing and extension for 1 min (temperature was different for each gene/primer pair), for 35 cycles and was performed in the commercial StepOne plus Real-time PCR system instrument (Applied Biosystems, Foster City, CA). No non-specific PCR products were found in any case. All reactions were performed in triplicate, and the data were normalized to a housekeeping gene, β-actin. PCR was repeated three times and the average of three experiments was used for gene expression analysis. After normalizing the expression of target gene to the expression of β-actin, the level of mRNA expression in each sample was expressed relative to the WT (*Fa/Fa*) control values.

**Table 1:** RT-PCR primer sequences.

Gene	5′-Primer (5′−3′)	3′-Primer (5′−3′)
ALT	TTCAAGCAGAGAGACAGGAG	TGAGGGAAGGAATACATGG
PEPCK	ATGTCAGAAGAGGACTTCGAGA	TGAATGGGATGACATACATGGT
FAS	GGACATGGTCACAGACGATGAC	GTCGAACTTGGACAGATCCTTCA
ACC alpha	TCGAAGAGCTTATATCGCCTATGA	GGGCAGCATGAACTGAAATTC
SREBP-1C	GGAGCCATGGATTGCACATT	AGGAAGGCTTCCAGAGAGGA
ChREBP	GATGGTGCGAACAGCTCTTCT	CTGGGCTGTGTCATGGTGAA
LPK	CGTTTGTGCCACACAGATGCT	CATTGGCCACATCGCTTGTCT
β-Actin	GCGCAAGTACTCTGTGTGGA	GACTCATCGTACTCCTGCTT

### Western blots

Snap frozen liver and tissue samples from four rats from each of the three experimental groups were lysed and protein was estimated by Bradford assay. 15 μg total protein was loaded in SDS-PAGE and transferred onto a polyvinylidene difluoride (PVDF) membrane. We detected PEPCK protein by incubating membrane with PEPCK antibody (Santa Cruz Biotechnology, Santa Cruz, CA) at room temperature for 1 hour. Actin was used as an internal loading control. Band intensities were quantified using NIH ImageJ software.

### Measurement of whole cell NAD+ and NADH levels

Whole cell NAD+ and NADH levels were measured from liver and tissue samples from three rats from each of the three experimental groups using a commercial colorimetric kit (BioVision Inc., Milpitas, CA), following the vendor’s instructions.

### Data analysis

Spectral peaks corresponding to HP [1-^13^C]pyruvate, [1-^13^C]lactate, and [1-^13^C]alanine were integrated over a 2 ppm range to generate dynamic curves for each metabolite. These curves were integrated over the entire time curve for the purpose of calculating metabolite ratios. To assess statistical differences between experimental groups (for both MR and rtPCR data), one-way analysis of variance (ANOVA) followed by Tukey’s multiple comparison post hoc testing was applied for each parameter between groups (GraphPad Software Inc., La Jolla, CA). Adipose tissue volume was estimated by applying a region-growing thresholding algorithm to the ^1^H SSFP images, which have a very bright fat signal, in OsiriX^60^.

## Electronic supplementary material

Supplementary figures and information
