# Supplementary material for: Non-invasive detection of divergent metabolic signals in insulin deficiency vs. insulin resistance in vivo
Source: Sci Rep. 2018 Feb 1;8:2088. doi: 10.1038/s41598-018-20264-w (PMC5794967; doi:10.1038/s41598-018-20264-w)
Supplement: Supplementary file 1 — Supplementary figures and information [file 41598_2018_20264_MOESM1_ESM.pdf]

Supplementary figures and information

Non-invasive detection of divergent metabolic signals in insulin deficiency vs. insulin resistance *in vivo*

Cornelius von Morze*, Prasanna K.R. Allu*, Gene Y. Chang, Irene Marco-Rius, Eugene Milshteyn, Zhen J. Wang, Michael A. Ohliger, Catherine E. Gleason, John Kurhanewicz, Daniel B. Vigneron, David Pearce

* These two authors contributed equally to this work.

Rat physiologic parameters

Select physiologic parameters for each experimental group are given in Figure S1. Weights by group at time of scan (one-way ANOVA summary statistics for weight: $p<0.0001$, $F=174.1$, $df=16$) were: *Fa/Fa* 287±19 g (mean±s.d.), *fa/fa* 357±15 g, WT-STZ 292±41 g. Blood glucose levels at time of scan (one-way ANOVA $p<0.0001$, $F=185.5$, $df=16$) were: *Fa/Fa* 169±18 mg/dL, *fa/fa* 577±57 mg/dL, WT-STZ 509±47 mg/dL. Adipose tissue volumes derived from ^{1}H MRI data (one-way ANOVA $p<0.001$, $F=27.21$, $df=7$) were: *Fa/Fa* 20±4 mL, *fa/fa* 105±25 mL, WT-STZ 16±3 mL.

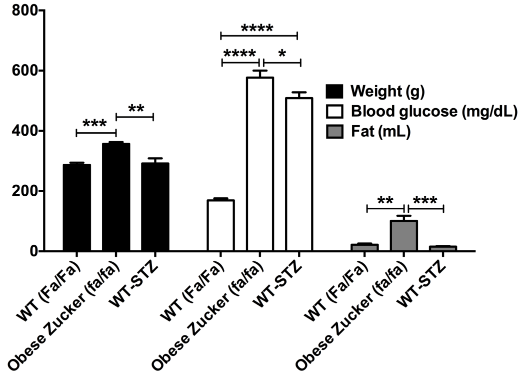

Figure S1. Basic physiologic parameters of Zucker rats used for MRI scans, as measured at time of MRI scan, by experimental group. Group means (WT n= 7, ZDF n= 6, WT-STZ n= 6) are shown with error bars indicating S.E.M. level. Statistically significant comparisons from one-way ANOVA with Tukey correction are labeled ($p<0.05$ *, $p<0.01$ **, $p<0.001$ ***, $p<0.0001$ ****).

Whole cell NAD⁺ and NADH measurements

Colorimetric assays of whole cell NAD^+ and NADH levels from liver and tissues of rats from each experimental group are presented in Figure S2.

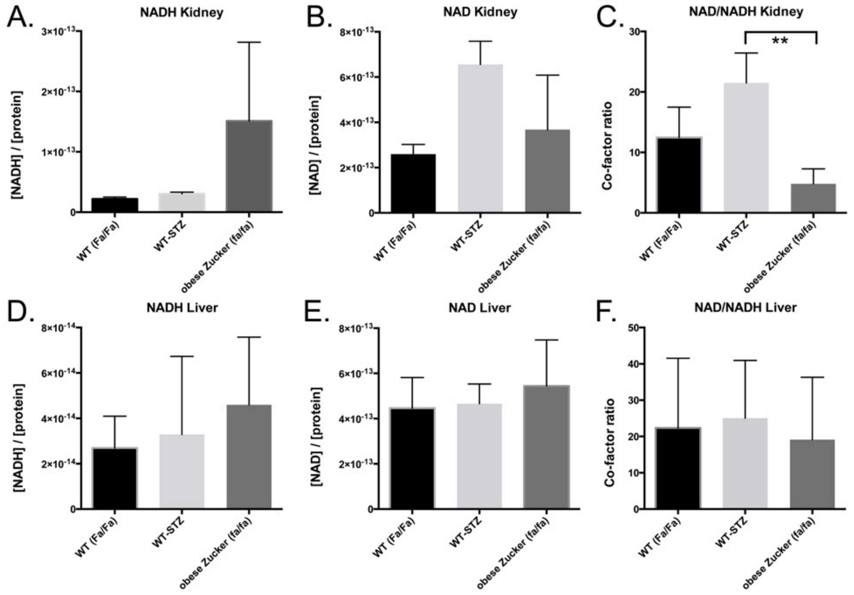

Figure S2. Whole cell NAD^+ and NADH measurements in kidney (A-C) and liver (D-F). Group means (WT n= 3, ZDF n= 3, WT-STZ n= 3) are shown with error bars indicating S.E.M. level. Statistically significant comparisons from one-way ANOVA with Tukey correction are labeled ($p<0.01$ **).

Addition of unlabeled α-ketoglutarate

Co-injection of equimolar unlabeled α-ketoglutarate along with HP [1-^{13}C]pyruvate produced no significant effects on HP [1-^{13}C]pyruvate spectra (Figure S3).

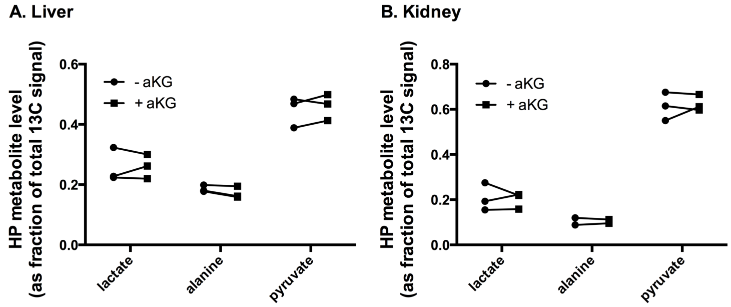

Figure S3. Effect of co-injection with HP [1-^{13}C]pyruvate of equimolar unlabeled α-ketoglutarate, on HP metabolites in liver (A) and kidney (B) of normal fed Sprague Dawley rats (n=3). Statistical comparison was by paired, two-tailed t-tests.

Western blots

Western blots for phosphoenolpyruvate carboxykinase (PEPCK) on tissues from all three groups were carried out as described under Methods, with results shown in Figure S4. Quantitation of densitometry data showed significant increases in PEPCK protein content of kidney of 24% (n.s.) and 49% (p<0.05) in kidney of WT-STZ and obese Zucker groups, respectively, as compared to WT. Increases of 58% (n.s.) and 75% (p<0.05) were detected in liver of WT-STZ and obese Zucker groups, respectively.

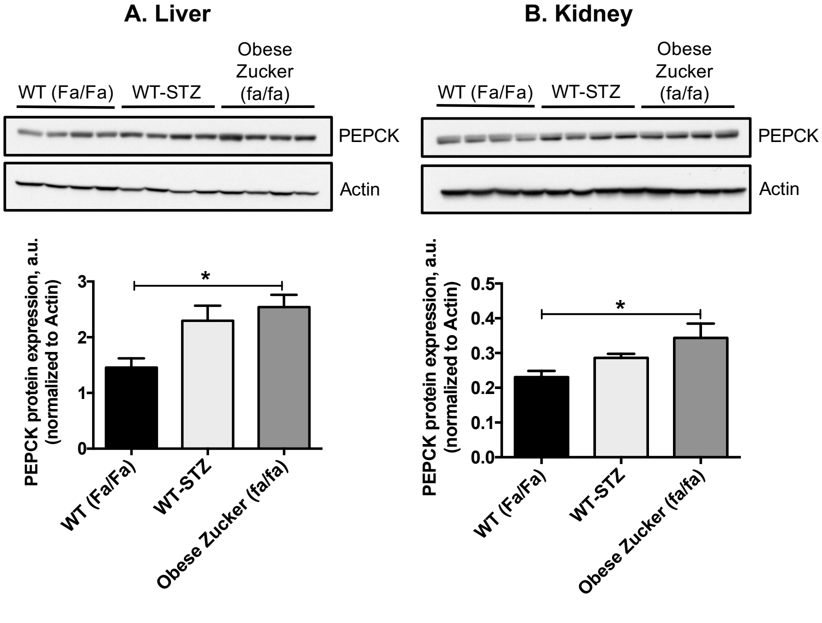

Figure S4. Western blots for PEPCK (with actin control) and corresponding quantification data for liver (A) and kidney (B) of rats from all three experimental groups. Group means (WT n= 4, ZDF n= 4, WT-STZ n= 4) are shown with error bars indicating S.E.M. level. Statistically significant comparisons from one-way ANOVA with Tukey correction are labeled (p<0.05 *).

Expression levels of LPK and ChREBP

RNA isolation and real time analysis for liver-type pyruvate kinase (LPK) and ChREBP from WT, obese Zucker, and STZ-treated WT animals were carried out as described under Methods. There were no significant differences in LPK or ChREBP expression profiles between these groups as depicted in Figures S5&S6.

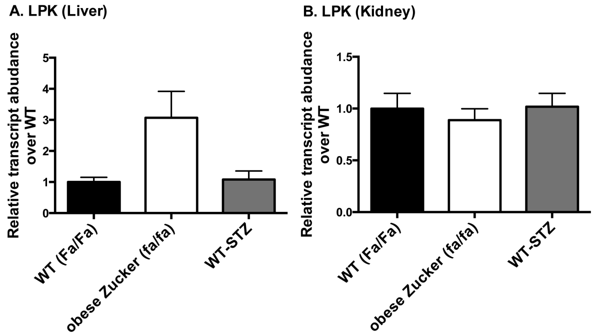

Figure S5. rtPCR measurements of liver-type pyruvate kinase (LPK) in liver (A) and kidney (B) of age and sex matched WT (*Fa/Fa*) (n=4), obese Zucker (*fa/fa*) (n=9), and WT-STZ (n=6) rats. The fold changes were measured relative to WT (*Fa/Fa*) and calculated by $2^{-\Delta Ct}$ method. The data were normalized to β-actin. All data represent mean+/-S.E.M. from three independent experiments. Statistical analysis was performed by one-way ANOVA with Tukey correction for multiple comparisons.

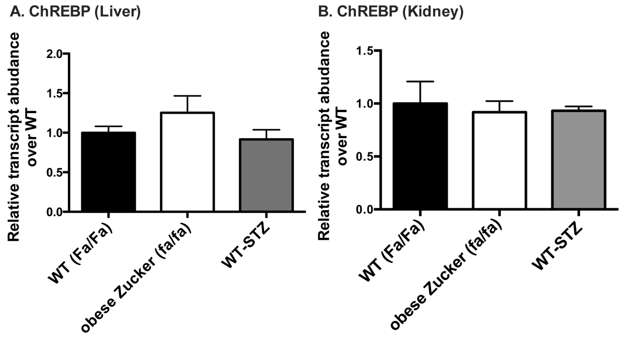

Figure S6. rtPCR measurements of ChREBP in liver (A) and kidney (B) of age and sex matched WT (*Fa/Fa*) (n=4), obese Zucker (*fa/fa*) (n=9), and WT-STZ (n=6) rats. The fold changes were measured relative to WT (*Fa/Fa*) and calculated by $2^{-\Delta Ct}$ method. The data were normalized to β-actin. All data represent mean+/-S.E.M. from three independent experiments. Statistical analysis was performed by one-way ANOVA with Tukey correction for multiple comparisons.
